# Crystal structures of the NO sensor NsrR reveal how its iron-sulfur cluster modulates DNA binding

**DOI:** 10.1038/ncomms15052

**Published:** 2017-04-20

**Authors:** Anne Volbeda, Erin L. Dodd, Claudine Darnault, Jason C. Crack, Oriane Renoux, Matthew I. Hutchings, Nick E. Le Brun, Juan C. Fontecilla-Camps

**Affiliations:** 1 Metalloproteins Unit, Institut de Biologie Structurale, CEA, CNRS, Université Grenoble-Alpes, 71, avenue des Martyrs, CS 10090, 38044 Grenoble cedex 9, France; 2 Centre for Molecular and Structural Biochemistry, School of Chemistry, University of East Anglia, Norwich Research Park, Norwich NR4 7TJ, UK; 3 School of Biological Sciences, University of East Anglia, Norwich Research Park, Norwich NR4 7TJ, UK

## Abstract

NsrR from *Streptomyces coelicolor (*Sc) regulates the expression of three genes through the progressive degradation of its [4Fe–4S] cluster on nitric oxide (NO) exposure. We report the 1.95 Å resolution crystal structure of dimeric holo-ScNsrR and show that the cluster is coordinated by the three invariant Cys residues from one monomer and, unexpectedly, Asp8 from the other. A cavity map suggests that NO displaces Asp8 as a cluster ligand and, while D8A and D8C variants remain NO sensitive, DNA binding is affected. A structural comparison of holo-ScNsrR with an apo-IscR-DNA complex shows that the [4Fe–4S] cluster stabilizes a turn between ScNsrR Cys93 and Cys99 properly oriented to interact with the DNA backbone. In addition, an apo ScNsrR structure suggests that Asn97 from this turn, along with Arg12, which forms a salt-bridge with Asp8, are instrumental in modulating the position of the DNA recognition helix region relative to its major groove.

## Abstract (web summary)

NsrR is a bacterial transcriptional regulator that acts as a nitric oxide (NO) sensor. Here, the authors present the crystal structure of NsrR, which reveals an unusual Fe-S cluster coordination and explains how NO exposure leads to the degradation of the cluster.

To counter the deleterious effects of the cytotoxin nitric oxide (NO), many bacteria, including several pathogens, have evolved mechanisms by which NO is sensed and a cellular response mounted^1^,^2^. The most widespread dedicated NO sensor regulator in bacteria is NsrR, a member of the Rrf2 superfamily of homodimeric transcriptional regulators^3^,^4^. NsrR from *Escherichia coli* (Ec) is known to regulate at least 60 genes^5^ including *hmp*, which encodes an NO detoxifying flavohaemoglobin (Hmp)^6^ that converts NO to nitrate (or nitrous oxide under anaerobic conditions). NsrR proteins from *Bacillus subtilis* (Bs) and *Streptomyces coelicolor* (Sc) have been shown to bind a [4Fe–4S] cluster, which functions as the NO sensing module^3^,^4^. In ScNsrR, reaction with NO was shown to result in loss of the iron–sulfur (Fe–S) cluster and formation of iron-nitrosyl species^7^. This process led to loss of high affinity DNA-binding and activation of genes that were previously repressed by [4Fe–4S] (holo-)ScNsrR, including those encoding Hmp-type NO detoxifying enzymes^3^,^8^.

The Rrf2 family of bacterial regulatory transcription factors is a functionally varied group, including, in addition to NsrR, regulators of iron-sulfur cluster biogenesis (IscR)^9^,^10^, cysteine metabolism (CymR)^11^, iron metabolism (RirA)^12^ and the newly-explored redox-sensitive regulator RsrR^13^. While CymR represents Rrf2 family regulators that do not have a co-factor, many contain three conserved Cys residues that have been shown, or are predicted, to bind either a [2Fe–2S] or a [4Fe–4S] cluster, dependent on the particular regulator. In all examples where data are available, the cluster functions as the sensory module and, therefore, plays a central role in the regulatory process.

EcIscR is known to contain a [2Fe–2S] cluster in its holo form^9^, and regulates transcription of more than 40 genes associated with iron-sulfur cluster biosynthesis^14^,^15^. IscR binds two different operator sequences termed type 1 and type 2. While only [2Fe–2S] IscR binds at type 1 sites, both holo- and apo-IscR can bind at type 2 sites. Recently, X-ray structures of apo-IscR from *E. coli*^16^ and *Thermincola potens*^17^ have been reported, together with apo-EcIscR in complex with DNA containing a type 2 site^16^,^17^. These structures reveal important information about the nature of the protein-DNA interaction, but a full understanding of the regulatory mechanism is currently hindered by the lack of structural information for the Fe–S cluster bound form.

We report here the 1.95 Å resolution crystal structure of holo-ScNsrR, the first for an Fe–S cluster-bound form of any Rrf2 superfamily regulator. Unexpectedly, Asp8 from one monomer of the ScNsrR homodimer is the fourth ligand to the [4Fe–4S] cluster further coordinated by three invariant Cys residues from the other. In addition, we find a connection between the cluster and a region next to the DNA-binding helix α3 mediated by a salt bridge between Asp8 and Arg12 with implications for NO-induced NsrR DNA binding modulation. NO binding studies of D8C and D8A ScNsrR variants and their comparison to wild type provide a rationale for the cluster Asp ligation. We also present the structure of a three-Cys-to-Ala (3CA) apo ScNsrR variant at 3.9 Å resolution. Taken together, our structures provide a solid basis to understand how this unusual cluster coordination conditions its degradation on NO exposure and how DNA-binding specificity is modulated. Furthermore, because of the similarity between NsrR and IscR (Fig. 1) our structural results also shed light on apo- versus holo-IscR structure/function relationships.

## Results

### Structure of the holo-ScNsrR homodimer

ScNsrR crystallized as a dimer spanning a distance of about 80 Å, with monomers being related by a crystallographic twofold axis. It shows an elongated fold that is typical of the Rrf2 family of transcriptional regulators, comprising eight α-helices and two anti-parallel β-strands (Fig. 2a). The X-ray structure parameters reveal high-temperature factors suggestive of a flexible and dynamic structure (Table 1). Nevertheless, the polypeptide chain was resolved for 137 of its 148 amino acid residues and forms two loosely interacting domains (residues 1–85 and 86–144, see Methods section).

A comparison with available related structures in the Protein Data Bank revealed that although the DNA-binding N-terminal domain is the most conserved one its conformation is highly variable. In terms of similarity, *Thermincola potens* apo-IscR^17^ is the closest one to the ScNsrR functional dimer giving a root-mean-square deviation of 2.22 Å for 214 out of a total of 274 superposed Cα atoms. Another similarity to other Rrf2 superfamily members is the highly hydrophobic nature of the ScNsrR dimerization interface (Supplementary Fig. 1), which buries a surface of about 2,050 Å^2^ per monomer. Putative DNA-binding regions in the two ScNsrR domains are discussed below.

Unique to the holo-ScNsrR structure is the presence of a [4Fe–4S] cluster that is ligated by three cysteine residues (Cys93, Cys99 and Cys105) from one monomer and Asp8 from the other (Fig. 2b). The latter forms a salt bridge with Arg12 from the same α1 helix, which also interacts with the carbonyl oxygen of Val36 of the DNA-binding helix-turn-helix (HTH) motif (Fig. 3). At the opposite side of the cluster Cys93 is exposed to solvent. The more buried Cys99 and Cys105 Sγ atoms are hydrogen-bonded to the main chain N atoms of Leu101 and Arg108, respectively. The position of Cys105-Sγ is further stabilized by the positive dipole moment of the α6 helix. Otherwise, the cluster environment is mainly hydrophobic, without H-bonds to the inorganic S atoms of the cluster. Anisotropic temperature factor analysis of the [4Fe–4S] cluster binding region at the dimer interface shows that the two monomers display orthogonally oriented agitation/disorder at this region (Supplementary Fig. 2). Holo-ScNsrR appears to be the first structurally characterized protein with a [4Fe–4S] cluster that is asymmetrically bound by ligands from two different subunits as indicated by our examination of all the other [4Fe–4S] cluster-containing protein structures found in the Protein Data Bank^18^. The observation of Asp8 as a ligand explains previous data from resonance Raman spectroscopy that revealed the presence of an oxygenic ligand in addition to three Cys residues^3^. In addition, data consistent with Glu85 being a cluster ligand^3^ can now be rationalized by its role in stabilizing the N-terminal region of ScNsrR (Supplementary Fig. 3).

### Structures of apo-ScNsrR homodimers

To better understand the role of the [4Fe–4S] cluster in DNA binding modulation we prepared and crystallized the 3CA apo-ScNsrR variant, analogous to the 3CA variants used previously for structural studies of EcIscR^16^ (see Methods section). The trigonal crystals of the variant displayed contact twinning with a [−*h* −*k* *l*] twin axis operator resulting in apparent hexagonal symmetry. They also had ∼62% solvent content and only diffracted to 3.9 Å resolution at the microfocus ID30-A3 beam line of the European Synchrotron Radiation Facility (Table 1 and Methods section). In spite of these limitations and because there were five ScNsrR monomers per asymmetric unit, we were able to solve its structure thereby gaining precious information about cluster-dependent DNA-binding modulation. Indeed, a comparison of the structures of wild-type holo and 3CA apo-ScNsrR shows that the [4Fe–4S] cluster conditions the conformation of two relevant regions. One of them is the C_93_EGDNPC_99_ sequence that forms a well-defined turn in the holo structure, but is less ordered in the apo structure where it adopts a different conformation (Fig. 2a and Supplementary Fig. 4); based on the superposition of the holo structure with that of the apo-IscR-DNA complex, the tip of this turn should contact the DNA backbone of the ScNsrR-binding site (Supplementary Fig. 5). Furthermore, Asn97 from the C_93_EGDNPC_99_ turn forms a H-bond with the carbonyl oxygen of Gly37, a residue situated in a turn next to the N terminus of the DNA recognition helix (RH) (Fig. 3a). In the apo structure this interaction is no longer possible (Fig. 3b). The other relevant change between the apo and holo ScNsrR structures is the disruption of the Fe-Asp8-Arg12-Val36 connection of the latter. In the apo structure the salt bridge between Asp8 and Arg12 appears to be preserved but the absence of the cluster changes the orientation of these residues causing the break of the H-bond between Arg12 and the carbonyl oxygen of Val36 (Fig. 3). The disruption of the Asn97-Gly37 and Arg12-Val36 H-bonds in apo-ScNsrR, indicated by the different conformation of the main chain in the medium-resolution apo structure (Fig. 3), results in the displacement of the RH by over 2 Å, which may be enough to prevent its binding to DNA, as observed.

### Properties of the S*c*NsrR D8A and D8C variants

Site-directed mutagenesis was carried out to test the importance of the Asp8 carboxylate [4Fe–4S] cluster ligand for DNA binding and, therefore, biological activity (Supplementary Fig. 6). D8A and D8C ScNsrR variants were prepared and isolated and were stable over a few weeks at 4 °C. Both variants were homodimeric. The D8C variant was almost fully loaded with a [4Fe–4S] cluster, as judged from its ultraviolet–visible absorbance spectrum (Supplementary Fig. 7a). Although the cofactor loading of the D8A mutant was lower (∼40%), UV-visible absorbance properties were also consistent with the presence of a [4Fe–4S] cluster (Supplementary Fig. 7c). To confirm the nature of the clusters bound in D8C and D8A variants, ESI-mass spectrometry under non-denaturing conditions was used^3^,^13^. The deconvoluted spectra (Supplementary Fig. 7b and d) revealed the presence of a [4Fe–4S] cluster, and minor amounts of other cluster species in both, demonstrating that, despite the loss of the natural aspartate ligand, a [4Fe–4S] cluster can still be assembled in the D8A and D8C variants.

The capacity of the variant proteins to bind the ScNsrR-regulated *hmpA1* promoter was tested by electrophoretic mobility shift assay experiments (Fig. 4 and Supplementary Fig. 8). For D8C ScNsrR, 50% binding of the DNA was observed at an approximately 20:1 cluster-bound protein to DNA strand ratio. For wild type ScNsrR, 50% binding was observed at a ratio<2:1 (Fig. 4 and ref. 3), demonstrating that replacement of Asp8 with Cys significantly reduced the affinity of ScNsrR for DNA. For D8A, there was no sign of DNA binding up to a ratio of 20:1 (Fig. 4). Thus, replacement of Asp8 with Ala abolished the regulator's affinity for DNA at physiologically relevant concentrations.

The [4Fe–4S]—containing ScNsrR D8A and D8C variants exhibited similar absorbance spectral changes to those for wild type when titrated with NO. In all cases nitrosylation resulted in loss of the 412 nm band and emergence of a new band at 357 nm, as previously reported for wild type ScNsrR^8^, indicating similarity in the final iron nitrosyl products formed^7^ (Supplementary Fig. 10a and b). Product formation was effectively complete at between 8 and 12 NO molecules per cluster, similar to that previously reported for the non His-tagged protein^8^. CD spectroscopy is particularly sensitive to differences in the cluster environment. The CD spectra of wild type and D8A [4Fe–4S] ScNsrR and the changes due to nitrosylation (Supplementary Fig. 10c) were similar to those previously reported for non-tagged ScNsrR^8^. In contrast, the initial CD spectrum of D8C [4Fe–4S] ScNsrR and the changes observed on nitrosylation were significantly different, with the greatest deviation observed at 2 NO per cluster (Supplementary Fig. 10c). Strikingly, though, the final spectrum closely resembled that of the other proteins, suggesting that the final nitrosylation product has a similar protein conformation. This could be interpreted as indicative of a different initial nitrosylation site on the cluster, consistent with the presumed similar lability of the four cysteine ligands. The overall reaction of the three [4Fe–4S] ScNsrR proteins with NO under pseudo-first order conditions was followed using stopped-flow absorbance spectroscopy, monitoring *A*_357 nm_ as a function of time (Supplementary Fig. 10d). While a full kinetic analysis will be reported elsewhere, a simple comparison of *A*_357 nm_ data over the first 10 s provides a means to assess the relative reactivity of the variants towards NO (Supplementary Fig. 10d). The data clearly show that the order of reactivity is D8A>wild type>D8C. The fourth iron of the D8A [4Fe–4S] cluster is likely bound by a labile solvent molecule, or some other loosely-associated small molecule anion, in the absence of a coordinating amino acid residue within binding distance. Such a ligand would be more labile than protein-bound thiolate or carboxylate ligands, and more readily displaced by NO. The greater sensitivity of wild type ScNsrR compared to D8C is also consistent with the greater lability of the coordinating carboxylate compared to a Cys residue^19^,^20^.

### Possible initial NO-binding site at the iron–sulfur cluster

Figure 5 shows a cavity map with a possible access path for NO, which is consistent with the data presented above. This path points at Asp8, the non-cysteinyl ligand to the [4Fe–4S] cluster. It is likely that the same access pathway at the dimer interface (Fig. 5) is used by exogenous DTT and CN^−^ to react with the unique iron ion of the [4Fe–4S] cluster while displacing Asp8 (refs 3, 4). Conversely, glutathione is too big to access via this channel and so does not bind the cluster^3^. The carboxylate group of Asp8 forms a monodentate bond with the unique iron of the cluster and a salt bridge/H-bond with Arg12 as discussed above (Fig. 3).

Besides providing a connection between the [4Fe–4S] cluster and the RH through Val36, this salt-bridge may weaken the carboxylate O–Fe bond thereby facilitating its cleavage, as required to form the first NO bond to this Fe. Indeed, in ferredoxins containing an [Fe–S] cluster Asp ligand, loss of the carboxylate-bound Fe is rather easily achieved^19^,^20^. The Asp8-Arg12 salt-bridge may also stabilize the α1 helix after dissociation of Asp8 from the cluster, as suggested by the likely preservation of this interaction in the apo-ScNsrR structure (Fig. 3b).

### Structural basis for IscR binding to type 1 or type 2 sites

The regulator IscR connects the ISC (for iron-sulfur cluster) and SUF (for sulfur assimilation) pathways of iron-sulfur cluster biosynthesis^21^. IscR can bind a [2Fe–2S] cluster and in its holo state, under iron sufficiency conditions, it represses the expression of the *isc* operon by binding to DNA at the type 1 IscR operator sequence. When the concentration of Fe–S clusters in the cell decreases, IscR loses its [2Fe–2S] cluster and it dissociates from the type 1 site. This derepresses the *isc* operon and stimulates Fe–S cluster biogenesis. IscR then reacquires a cluster and re-binds to the type 1 site. Conversely, if low iron levels are coupled to oxidative stress (aerobic) conditions the resulting apo form of IscR binds to a different DNA site called type 2 where it elicits Fe–S cluster biogenesis by activating the *suf* operon which encodes the SUF pathway, a process that is also influenced by other regulators. Thus, IscR is capable of regulating DNA expression in both its holo and apo forms.

Crystal structures of apo IscR dimers alone and in complex with the type 2 regulatory site are available (Fig. 6a)^16^,^17^. They have shown that apo IscR is already well poised to bind DNA at that site because the differences between its bound and unbound conformations are small. Ser40, Glu43 and Gln44, which are residues contained in the IscR RH, bind the major groove of the type 2 DNA sequence. Importantly, Glu43 forms a bidentate interaction with the amino groups of the C7C8 and C7′C8′ bases. Although the E43A mutation did not have a major influence in the DNA binding affinity of IscR it had a marked effect on the specificity for type 1 and 2 sites: native apo-IscR does not bind to type 1 sites while its E43A variant does^16^. In ScNsrR the counterparts of EcIscR Ser40, Glu43 and Gln44 are Thr41, Ala44 and Lys45. The differences in residue type are consistent with the different DNA sequences the two regulators bind^3^.

Whereas the type 2 DNA site has C7C8 bases on one side and C7′C8′ bases on the other, the type 1 DNA site contains C7C8 bases on one side but T7'T8' bases on the other (see Fig. 6 and ref. 16). The latter bases should have a methyl moiety rather than a polar group pointing at the major groove. Consequently, when positioned as in the apo-IscR-DNA complex, the carboxylate group of Glu43 in one of the IscR monomers would not be able to interact with the T7′T8′ bases. This observation provides a rationale for both the observed lack of binding of apo-IscR at the type 1 site and the observed binding of its E43A variant to that site^16^.

**Figure 1:**
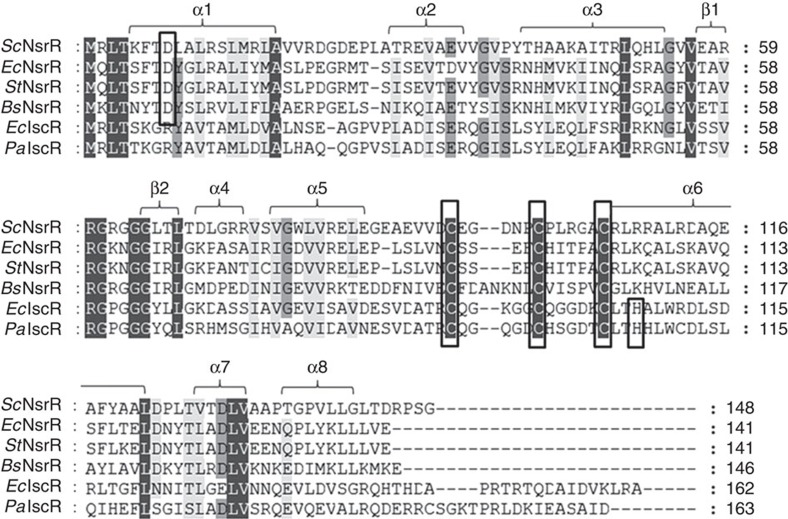
Amino acid alignment of *S. coelicolor* NsrR with related proteins. Other shown NsrRs are from *E. coli* (EcNsrR), *Salmonella enterica typhimurium* (StNsrR) and *B. subtilis* (BsNsrR). IscR proteins are from *E. coli* (EcIscR) and *P. aeruginosa* (PaIscR). The alignment was carried out using Clustal Omega^45^ and presented using Genedoc^46^. Secondary structural elements of ScNsrR are indicated above the sequences. Residues that serve as cluster ligands in NsrR and IscR are indicated by boxes. Black colour indicates totally conserved residues, grey indicates highly conserved residues, and light grey indicates residues that are either well conserved or conservatively substituted.

**Figure 2:**
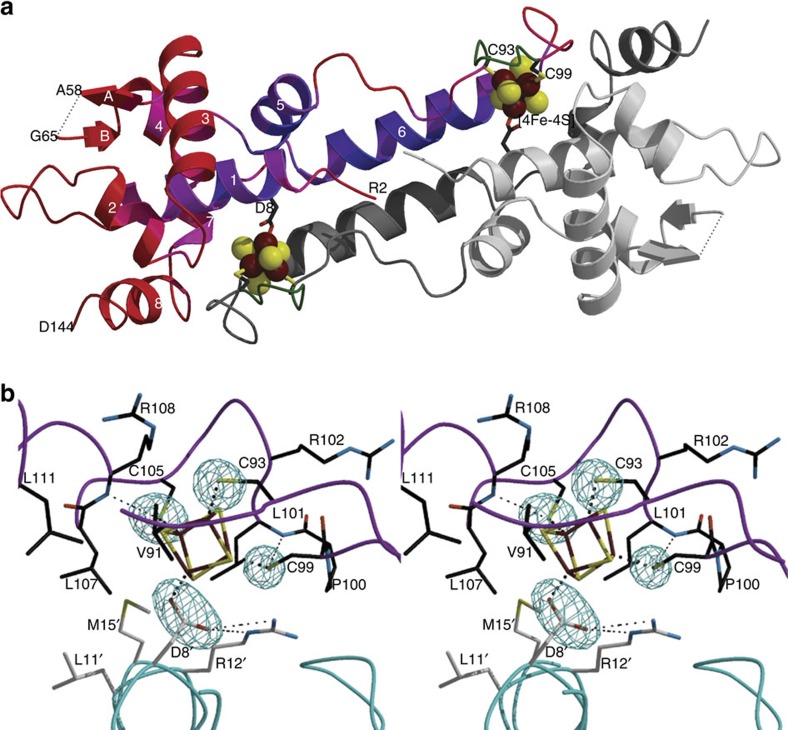
Structure of holo ScNsrR. (**a**) Ribbon depiction of the functional dimer. One monomer is coloured from blue to red according to increasing temperature factors (from 56.5 to 97.4 Å^2^) and the other monomer has its two domains depicted in light and dark shades of grey (see Methods section). Secondary structure elements are labelled 1–8 for α-helices and A–B for β-strands. Fe and S atoms are shown as brown and yellow spheres, respectively. The functionally important C93–C99 loop (see text) is coloured green. (**b**) Stereo view of the [4Fe–4S] cluster environment with omit electron density peaks (blue mesh, contoured at six times the r.m.s. σ level of the map) corresponding to the Sγ and carboxylate ligands. The main chains of the two monomers are depicted in pink and blue, respectively; Fe-ligand and H-bonding interactions are indicated with thick dotted and thin dashed lines, respectively. Atom colours are: O red, N blue and C black or grey.

Understanding the structural basis for the modulation of IscR binding specificity as a function of the presence or absence of a [2Fe–2S] cluster has been hampered by the lack of a holo-IscR structure (or of any other FeS-binding member of the Rrf2 family of transcriptional regulators). Our holo- and apo-ScNsrR structures shed considerable light on the way Fe–S cluster coordination could alter both DNA binding affinity and specificity in both IscR and NsrR. As mentioned above, the C_93_EGDNPC_99_ sequence (Fig. 1) forms a well-defined turn in the holo-ScNsrR, which is stabilized by the [4Fe–4S] cluster. The corresponding sequence in EcIscR (Fig. 1) is C_92_QGKGGC_98_ with Lys95 being both suitable and properly positioned to interact with the phosphate group of A15 (ref. 22) (Fig. 6c). H-bonding by Asn97 from the C93–C99 turn in holo-ScNsrR, and Arg12 appear to stabilize the α3 RH at about 2.5 Å from its counterpart in the superposed apo-IscR-DNA complex (Fig. 6b,c). If holo-EcIscR forms a similar turn with its C_92_QGKGGC_98_ sequence, an equivalent displacement of the RH, possibly involving Gln35, would result in the carboxylate group of Glu43 being displaced away from that sequence, explaining why it can bind the type 1 site with its T7′T8′ sequence. Glu43 would instead approach the base at position 6 that, according to sequence analysis of type 1 sites (see Supplementary Information in ref. 16), is either C or G. Both these bases could form a monodentate H-bond with Glu43. The 3CA-ScNsrR apo structure shows that the conformation of the C_93_EGDNPC_99_ loop depends on the presence or absence of the Fe–S cluster (Figs 2a and Supplementary Fig. 4).

Similar conformations and positions of the ScNsrR C93-C99 and the EcIscR C92-C98 turns require similar ligation of the two Cys thiolates to the corresponding Fe–S clusters within an equivalent protein cavity. Because holo-ScNsrR contains a cuboid [4Fe–4S] cluster and holo-IscR a rhomboid [2Fe–2S] cluster, maintaining these two thiolate ligations at equivalent positions would require different orientations for the remaining two ligands (Cys105 and Asp8 in ScNsrR and Cys104 and His107 in EcIscR; Fig. 1). Examination of the IscR structure indicates that a shift in the 105–123 helix to a position similar to the one occupied by its counterpart in holo-ScNsrR would allow for [2Fe–2S] cluster coordination while keeping Cys92 and Cys98 in positions similar to the ones occupied by ScNsrR Cys93 and Cys99. Preservation of two Cys positions in the conversion of a [2Fe–2S] cluster to a [4Fe–4S] cluster was observed with the non-conserved cluster of the FeFe-hydrogenase maturase HydE^23^. The presence of the flexible and long His107 side chain in EcIscR is likely to allow for [2Fe–2S] cluster coordination because of its adaptability as a ligand. Indeed, the H107C mutation resulted in only partial anaerobic repression of P_iscR_-*lacZ* and no formation of holo-IscR was observed *in vitro* for this variant^24^. In contrast, the ScNsrR D8C variant reported here was isolated almost replete with a [4Fe–4S] cluster.

## Discussion

**Figure 3:**
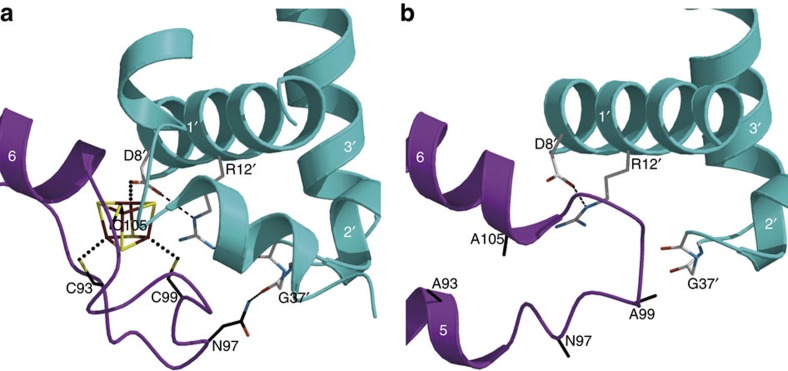
Structural changes induced by the loss of the FeS cluster. (**a**) In holo ScNsrR Arg12 connects the Asp8 [4Fe–4S] cluster ligand with Val36 located next to the DNA-recognition helix (α3′ RH). In addition, Asn97 from the other monomer establishes an H-bond with the carbonyl oxygen from Gly37. (**b**) The absence of the cluster in apo 3CA-ScNsrR causes the disruption of the Arg12-Val36 and Asn97-Gly37 H-bonds, which results in a shift of about 2.0 Å of each of the ScNsrR homodimer RHs.

We have succeeded in solving the crystal structures of (i) holo ScNsrR, the first for a member of the Rrf2 family of transcription regulators with a bound Fe–S cluster and (ii) the apo 3CA-variant of ScNsrR. Comparison of holo-ScNsrR with apo IscR shows that one of the consequences of Fe–S cluster binding is the structuration of the sequence contained between Cys93 and Cys99 (ScNsrR numbering), which then forms a well-defined turn. This region is disordered in apo IscR and adopts a different conformation in apo 3CA-ScNsrR (Fig. 2a and Supplementary Fig. 4). The C_93_EGDNPC_99_ turn includes Asn97, a residue that in holo ScNsrR interacts with the carbonyl oxygen of Gly37 (Fig. 3a). Furthermore, Fig. 1 shows that the most variable sequence region when comparing this family of regulators corresponds to this turn. In addition, the superposition of apo IscR with holo ScNsrR (Fig. 6c and Supplementary Fig. 5) shows that EcIscR Lys95 could also be a DNA backbone ligand^22^. Another interesting case is BsNsrR, whose turn sequence contains a two-residue insertion relative to its *S. coelicolor* counterpart, and has one Lys residue as well. Like IscR, BsNsrR can bind DNA in both its dimeric apo and holo forms^4^,^25^, while ScNsrR was shown to bind to different gene promoters depending on the extent of the cluster reaction with NO^8^. Three-dimensional superposition of holo-*Sc*NsrR with IscR-DNA complexes and our 3CA-ScNsrR variant, along with amino acid sequence comparisons, suggest that Asn97 from the turn between Cys93 and Cys99 helps to position the RH region when bound to the major groove of its cognate DNA sequence. It is likely that the structural factors that control IscR binding to type 1 and 2 sites in DNA play an equivalent role in ScNsrR. Taken together, these findings provide a rationale for the modulation of DNA binding as a function of cluster integrity in both NsrRs and IscRs.

Specific to ScNsrR function is the inter-monomer ligation to the [4Fe–4S] cluster by Asp8. It not only represents a novel structural motif for Fe–S proteins in general, with a cluster coordinated by a non-cysteinyl ligand from another monomer, it also sheds considerable light on the NO-based mechanism of cluster degradation. Indeed, the ScNsrR structure is consistent with the idea that the breaking of both inter-monomer Asp8-[4Fe–4S] bonds, caused by their substitution with NO, will initiate both cluster degradation and structural changes. These changes are very likely responsible for the observed modulation of promoter binding affinity by the gas^8^. Importantly, NO binding studies indicate that the nature of the ligand, either Asp or Cys, or its absence in the D8A variant, conditions the reaction of the cluster with the gas (Supplementary Fig. 7). This result is consistent with a cavity map that suggests the existence of a path for NO diffusion at the ScNsrR surface, next to the Asp8 position between the two monomers (Fig. 5).

**Figure 4:**
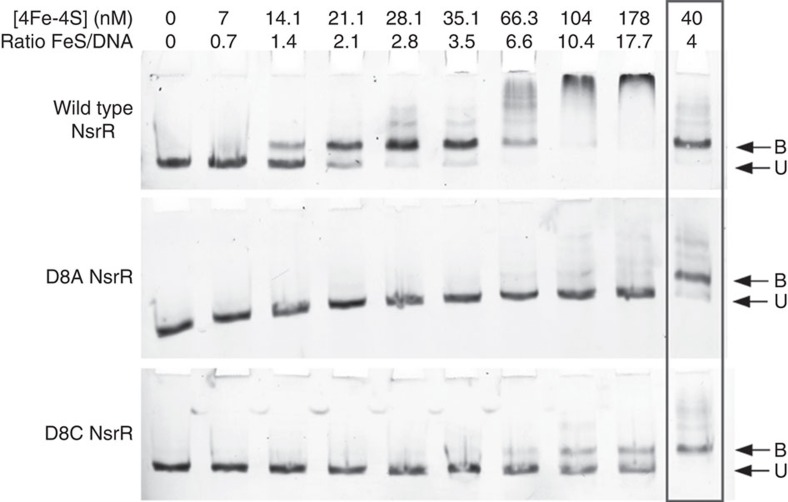
EMSA analysis of DNA binding by ScNsrR variants. Compared are the binding affinities of the wild type protein and its D8A and D8C variants to the ScNsrR-specific DNA promoter *hmpA1* site. The D8A variant displayed no affinity to this site while the D8C variant has lowered affinity. Box: reference sample of wild type ScNsrR. The [4Fe–4S] protein concentration was determined by UV-visible spectrometry. See also Supplementary Fig. 8. B, bound; U, unbound.

Site-directed replacement of ScNsrR Asp8 with either Ala or Cys had a clear impact on DNA binding affinity (Fig. 4). The alanine side chain cannot bind the [4Fe–4S] cluster, which consequently generates a large proportion of ScNsrR lacking this cofactor; this notion is reinforced by the fact that neither the Ala variant nor the apo protein bind DNA at physiologically relevant concentrations. Conversely, an inter-monomer bridging Cys ligand like in the D8C variant should form a strong bond with the unique iron of the cluster, which accounts for the high proportion of holo-ScNsrR obtained with this variant. Furthermore, the very significant drop in DNA binding affinity observed for the D8C variant can be explained by the absence of a bonding interaction between Cys8 and Arg12, which, in turn, disrupts the connection of the [4Fe–4S] cluster with the short turn preceding the α3 RH discussed above for the native protein.

The question as to why IscR and NsrR, two Fe–S cluster-binding members of the Rrf2 family, do not coordinate the same type of cluster *in vivo* might be answered by considering the nature of their effectors. Sensing and responding to either cluster availability/ROS or NO may require dissimilar cluster degradation/reaction processes^7^ which will also cause different modifications in protein structure. From a structural point of view, accommodating a [4Fe–4S] cluster rather than a [2Fe–2S] cluster within a similar protein cavity is possibly also facilitated by the provision in ScNsrR of ligation from the other monomer.

In summary, our structures of ScNsrR strongly suggest that one of the main effects of cluster binding is the stabilization of an inter-cysteine turn that binds to the DNA backbone modifying in the process the orientation of the α3 RH at the major groove of the nucleic acid. The other major determinant of the α3 RH orientation is Arg12 which, along with Asp8, significantly changes its position after cluster loss. Further studies will involve the preparation and structural and functional analyses of variants at the ScNsrR C_93_EGDNPC_99_ sequence as well as complexes of both native and variant proteins with cognate DNA sites.

## Methods

### Purification of *Streptomyces coelicolor* NsrR variants

All plasmids were purchased from Genscript (Supplementary Methods). C-terminally His-tagged ScNsrR proteins (wild type [UniProt accession code: Q9L132] and variant proteins D8A, D8C and C93A/C99A/C105A or 3CA) were overexpressed in *E. coli* BL21DE3 (New England Biolabs Inc). Briefly, bacterial cultures were grown at 37 °C, in LB broth supplemented with the appropriate antibiotic, until A_600 nm_ reached 0.6–0.8 (ref. 3). Cultures were then cold shocked on ice for 18 min. Protein overproduction was initiated by the addition of 10 μM isopropyl β-D-thiogalactopyranoside and cultures incubated at 30 °C. After 50 min, cultures were supplemented with 200 μM ferric ammonium citrate, 50 μM L-methionine and incubated for a further 3.5 h (ref. 26). Cells were collected by centrifugation, washed with buffer A (50 mM Tris, 5% (v/v) glycerol, 100 mM NaCl pH 8.0), and stored at −80 °C until needed.

Protein purification and handling was carried out under strictly anaerobic conditions (O_2_≤2 p.p.m.) in a glove box (Belle Technology) unless otherwise stated. Briefly, cells were resuspended in anaerobic buffer A and disrupted by sonication outside of the glovebox. The cell lysate was clarified by centrifugation in sealed anaerobic tubes outside of the glovebox, 40,000 × g for 45 min at 1 °C. The supernatant was loaded on to a HiTrap Ni^2+^ chelating column (2 × 5 ml; GE Healthcare Life Sciences) previously equilibrated with buffer A, and washed with 5% (v/v) buffer B (50 mM Tris, 5% (v/v) glycerol, 100 mM NaCl, 200 mM L-histidine, pH 8.0). Bound proteins were eluted using a linear gradient from 5 to 50% (v/v) buffer B. Fractions containing NsrR were pooled and loaded directly onto a HiTrap Heparin HP column (2 × 1 ml; GE Healthcare Life Sciences), washed with buffer A, and eluted with buffer C (50 mM Tris 5% (v/v) glycerol, 2 M NaCl, pH 8.0). Fractions containing NsrR were pooled and stored in an anaerobic freezer until needed^3^. The [4Fe–4S] cluster-containing protein concentration was determined using an extinction coefficient of ɛ_406 nm_=13.30 (±0.19) mM^−1^ cm^−1^ (ref. 3) and apo-protein concentrations were determined using the method of Smith (Pierce)^27^ with bovine serum albumin as standard. Ultraviolet–visible absorbance measurements were made with a Jasco V500 spectrometer.

### *Sc*NsrR NO reactivity measurements

Ultraviolet–visible absorbance measurements were made with a Jasco V500 spectrometer and CD spectra were measured with a Jasco J810 spectropolarimeter. All spectroscopic titrations were performed anaerobically in a septa-capped 1 cm quartz cuvette using a buffer system of 50 mM Tris, pH 8.0, 1 M NaCl, 5% (v/v) glycerol, 0.03 mM GSH, and 1 mM Arg to protect against protein precipitation. The NO-generating compound proliNONOate (Cayman Chemicals) was introduced to protein as aliquots of a stock solution in 25 mM NaOH solution. Independent determination of NO release kinetics of the NONOate in the titration buffer system was used to calculate the amount of NO present at any time in titration solution. All solutions used for titrations were stored and manipulated inside an anaerobic glovebox (Belle Technology). The somewhat higher stoichiometry of reaction observed here for the His-tagged proteins compared to that previously observed for non-tagged NsrR^8^ is attributed to a minor unknown side reaction that resulted in a small amount of insoluble product.

UV-visible stopped-flow experiments were performed with a Pro-Data upgraded Applied Photophysics Bio-Sequential DX.17MVspectrophotometer, with a 1 cm path length cell. Absorption changes were detected at a single wavelength (357 nm). Before use, the stopped-flow system was flushed with 30 ml of anaerobic assay buffer and experiments were carried out using gas tight syringes (Hamilton). All solutions used for stopped-flow experiments were stored and manipulated inside an anaerobic glovebox (Belle Technology). Fitting of kinetic data at 357 nm to single exponential functions was performed using Origin (version 8, Origin Labs).

### Electrophoretic mobility shift assays

Fluorescently (6-FAM) labelled DNA fragments carrying the *hmpA1* (SCO7428) promoter were PCR amplified using 6-FAM labelled primers (Supplementary Fig. 8) with *S. coelicolor* genomic DNA as the template^3^. DNA fragments were extracted/purified using QIAquick gel extraction kit (Qiagen) according to the manufacturer's instructions and quantitated using a nanodrop ND2000c spectrometer (Thermo Scientific). OligoCalc was used to calculate the molecular weight of the double stranded 6-FAM labelled probes^28^. Bandshift reactions (20 μl) were carried out in binding buffer (10 mM Tris, 54 mM KCl, 0.3% Glycerol, 1.32 mM glutathione, pH 7.2). Briefly, 1 μl of DNA was titrated with aliquots of ScNsrR (or variants) to a ∼18-fold molar excess, and incubated on ice for ∼10 min. Loading dye (2 μl, containing 0.3% (w/v) bromophenol blue) was added and the reaction mixtures were immediately separated on a 5% (w/v) polyacrylamide gel using a Mini Protean III system (BioRad) running at 30 mA for 30 min in 1 × TBE (89 mM Tris, 89 mM boric acid, 2 mM EDTA). Gels were visualised (Ex_473 nm_, Em_510 nm_) on a Typhoon FLA 9500 (GE Healthcare Life Sciences) (see Fig. 4 and Supplementary Fig. 9).

### ESI-mass spectrometry of cluster bound ScNsrR

His-tagged wild type, D8A or D8C ScNsrR was exchanged into 250 mM ammonium acetate pH 8.0 using Zeba spin desalting columns (Thermo scientific), diluted to ∼25 μM cluster and infused directly (0.3 ml h^−1^) into the ESI source of a Bruker micrOTOF-QIII mass spectrometer (Bruker Daltonics, Coventry, UK) calibrated with ESI-L low concentration tuning mix in the positive ion mode (Agilent Technologies, California, USA). Full mass spectra (*m/z* 500–3500) were recorded for 5 min with parameters as follows: dry gas flow 4 l min^−1^, nebuliser gas pressure 0.8 Bar, dry gas 180 °C, capillary voltage 2,750–4,500 V, offset 500 V, ion energy 5 eV, collision RF 200 Vpp, collision cell energy 10 eV. Spectra were combined, processed using the ESI Compass 1.3 Maximum Entropy deconvolution routine in Bruker Compass Data analysis 4.1 (Bruker Daltonik, Bremen, Germany). Exact masses are reported from peak centroids representing the isotope average neutral mass. For apo-proteins, these are derived from *m/z* spectra, for which peaks correspond to [M+nH]^n+^/n. For cluster-containing proteins, where the cluster contributes charge, peaks correspond to [M+FeS^x+^+(n−x)H]^n+^/n, where M is the molecular mass of the protein, FeS is the mass of the iron-sulfur cluster of x+ charge, H is the mass of the proton and n is the total charge. In the expression, the x+ charge of the cluster offsets the number of protons required to achieve the observed charge state (n+)^29^. Predicted masses are given as the isotope average of the neutral protein or protein complex, in which cofactor-binding is expected to be charge compensated.

### ScNsrR crystallization

*Native holo-ScNsrR:* initial crystallization trials were carried out using a Gryphon robot (Art Robbins Inst. CA, USA) inside a customized anaerobic glove box, 96-well plates and 13 commercial kits. A total of 1,248 conditions were thus rapidly explored. In each experiment, 200 nl of a solution containing 16.4 mg ml^−1^ of ScNsrR in 50 mM Tris Base, pH 8.0, 300 mM NaCl and 5% v/v glycerol were mixed with 200 nl of each commercial crystallization solution. The plates were manually sealed and the sitting drops were equilibrated against 100 μl of the latter solution at 20 °C. A rod-shaped brownish crystal grew after a few days from a commercial condition using 4 M NaCl, 100 mM HEPES buffer, pH 7.0. This crystal was mounted directly from the original drop in a cryo-loop after adding an excess of cryo-protecting solution prepared adding 20% v/v glycerol to the reservoir solution. The crystal was then flash-cooled in liquid propane inside the glove box,^30^ and stored in liquid nitrogen. Anaerobic crystallization was subsequently scaled up using hanging drops made by mixing 1 μl protein solution with 1 μl crystallization solution equilibrated against 1 ml of the latter. Optimal ScNsrR crystallization conditions from these experiments were found to be 3.3 to 3.9 M NaCl, 100 mM HEPES pH 7.3 to 7.9 and 7.2 mM tetraethyleneglycol monomethyl ether (crystals 1 and 2 in Table 1).

*3CA-ScNsrR:* Initial crystallization trials were carried out using a Gryphon robot as described in the previous paragraph. Colourless crystals grew as fine needles after a few days from a commercial condition using 10% v/v 2-methyl, 2,4-pentanediol, 100 mM Tris buffer, pH 8.0. Crystallization assays were subsequently scaled up using hanging drops made by mixing 1 μl protein solution with 1 μl crystallization solution equilibrated against 1 ml of the latter. Optimal 3CA-ScNsrR crystallization conditions from these experiments were found to be 10% v/v 2-methyl, 2,4-pentanediol, 100 mM Tris buffer, pH 8.6 (crystal 3 in Table 1). A slightly different crystal form was obtained using 9% PEG 6000 with 100 mM Tris buffer at pH 8.1 (crystal 4 in Table 1).

### X-ray structure determination and structural analyses

All flash-cooled ScNsrR crystals were kept under a cold (100 K) N_2_ stream during X-ray data collection. The presence of Fe was confirmed by an X-ray absorption spectrum, measured with a Roentec X-Flash multichannel analyzer at beamline BM30a of the European Synchrotron Radiation Facility (ESRF) in Grenoble, France. The X-ray wavelength providing the maximum iron anomalous signal was determined to be 1.7414 Å with the program CHOOCH^31^. Subsequently, highly redundant 2.6 Å resolution X-ray diffraction data were collected at this wavelength from a single crystal with an ADSC Quantum 315r detector at the same beamline (Table 1, first column). These and all subsequent data obtained from other crystals were processed with the XDS package^32^) and scaled with the AIMLESS program^33^ of the CCP4 package^34^. Next, we used the PHENIX program package^35^ with these data to locate individual Fe sites of the [4Fe–4S] cluster with a Hybrid Substructure Search, to obtain single anomalous dispersion (SAD) phases with a figure of merit of 0.321 by refining the Fe parameters and using density modification for further phase improvement. During this process the space group was assigned as P6_5_22. The resulting phases were of sufficient quality to obtain an interpretable 2.6 Å resolution electron density map that allowed us to trace most of the polypeptide chain with the program COOT^36^.

Data were collected to 1.95 Å resolution from another crystal, at a remote wavelength of 0.984 Å, with a Dectris Pilatus 6M-F detector (Table 1, second column) at ESRF beamline ID23. These data were processed and scaled as described above and used for model refinement. Initial positional, TLS and individual B-factor refinement was done with the program phenix.refine^37^, which automatically determined two TLS groups corresponding to residues 2-85 and 86-144 to model anisotropic motions of these two domains. Final refinement was performed with the program Refmac5 (ref. 38). Refinement cycles were alternated with manual corrections using COOT. No outliers in the Ramachandran plot were found for the refined model consisting of residues 2-58 and 65-144. However, due to a high degree of disorder in the structure, several exposed loops and residues are poorly defined in the electron density map. Supplementary Fig. 11 shows the final electron density map obtained for the DNA-binding α-helix 3.

The best crystal of the triple 3CA (C93A/C99A/C105Q) apo-ScNsrR variant provided diffraction data to 3.9 Å resolution collected at a wavelength of 0.9677 Å with an Eiger 4M detector at ESRF beamline ID30-A3. These data were assigned by XDS to a trigonal space group. Starting from the refined structure of a holo-ScNsrR monomer, a molecular replacement solution was found by the program PHASER^39^, which identified the presence of 5 apo-ScNsrR copies in the asymmetric unit and determined the crystal symmetry to be P3_2_21 (Table 1, third column). In the trigonal crystal apo-ScNsrR molecules pack as a wide cylinder around a large internal solvent region of about 100 Å diameter. Four of the five apo 3CA-ScNsrR molecules form two non-crystallographic symmetry (ncs)-related dimers while the 5th one forms a third dimer with a neighboring monomer related by twofold crystallographic symmetry. The validity of the found molecular replacement solution was confirmed by the structural similarity of all three dimers to the functional holo-ScNsrR dimer. Another crystal of the triple CA variant had a significantly larger unit cell and gave diffraction data to 4.0 Å resolution (Table 1, fourth column). Although the indexing procedure of XDS suggested a hexagonal space group, a molecular replacement solution could only be found by PHASER when imposing P3_2_21 crystallographic symmetry, using two dimers and one monomer of the structure of holo-ScNsrR as starting models. Subsequent verification of the data by phenix.xtriage^35^ suggested the presence of a significant amount of twinning for both apo-ScNsrR crystals with a [−*h*, −*k*, *l*] twofold twin operator, in agreement with the apparent hexagonal symmetry found by XDS. Rigid body refinement was started for secondary structure elements for both apo 3CA-ScNsrR crystals, followed by tightly ncs-restrained positional and temperature (B-) factor refinement using Refmac5. Next the electron density maps were greatly improved by density modification with 10-fold multi-crystal averaging and solvent flattening using crystals 3 and 4 with PHENIX, alternating with extensive manual model corrections with COOT. Continued positional refinement was performed with PHENIX using torsion ncs restraints, automatically determined TLS groups and grouped B-factors. This was again alternated with manual corrections with COOT. For the final refinement cycles the [−*h*, −*k*, *l*] twin operator was included, indicating 14 and 34% twinning in crystals 3 and 4 (Table 1), respectively. In addition, one TLS group was now defined for each apo-NsrR molecule and secondary structure restraints were added. This strategy yielded good refinement statistics for a 3.9 Å resolution structure (Table 1, column 3) with no outliers in the Ramachandran plot. The resulting apo 3CA-ScNsrR model, consisting of residues 2–59 and 66–134, is much more disordered than its holo counterpart, as indicated by an average calculated B-factor of more than 200 Å^2^. Supplementary Figure 12 illustrates how this structure fits to the density-modified map obtained after five cycles of a final 10-fold multi-crystal averaging and solvent flattening procedure.

**Figure 5:**
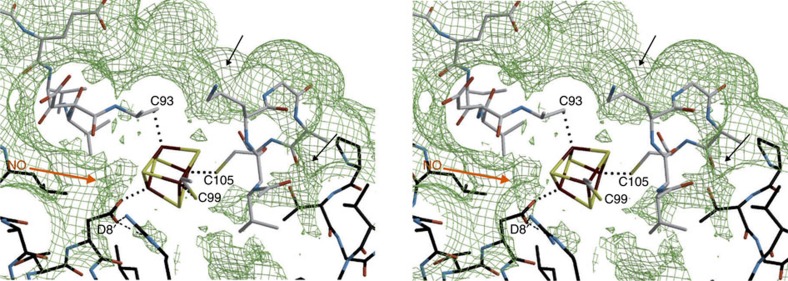
Stereo view of the FeS cluster environment in ScNsrR. A cavity map calculated with a probe radius of 0.6 Å is shown as a green mesh. The orange arrow shows a likely pathway for NO diffusion into the protein. Other plausible access routes are indicated by small black arrows. Atoms are coloured as in Figs 2b and 3. Cluster bonds and H-bonding interactions of Asp8 and Arg12 are shown as dotted and dashed lines.

**Figure 6:**
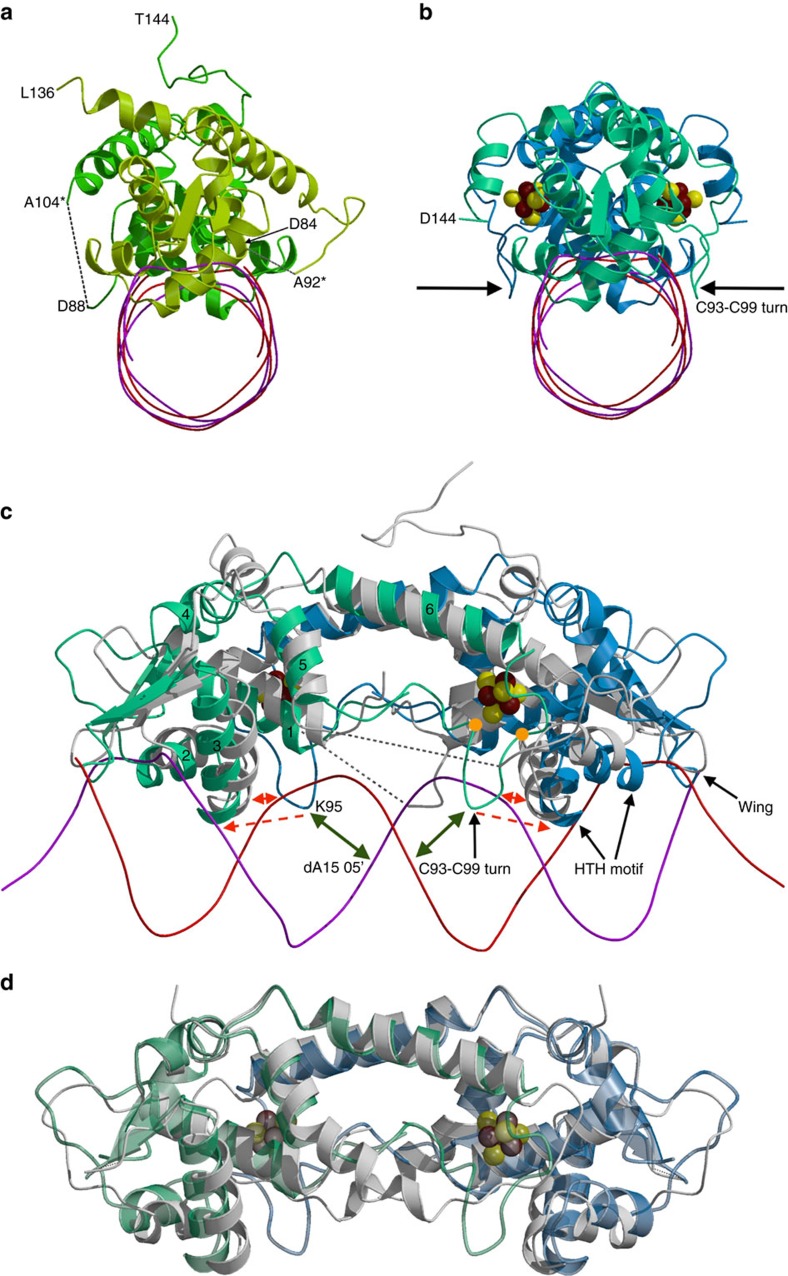
Comparison of apo-IscR-DNA complex, holo- and apo-ScNsrR. Ribbon models of (**a**) the apo-IscR-DNA complex, (**b**) holo-ScNsrR superposed with the DNA part of apo-IscR-DNA, (**c**) a perpendicular view of the superposition and (**d**) superposition of holo- and apo 3CA-ScNsrR. In **a** A92* and A104* correspond to Cys residues in the native IscR protein. The black arrows in **b** point at the C_93_EGDNPC_99_ turn in holo-ScNsrR. The corresponding region in IscR, shown as dotted lines between residues 84-92 and 88-104 in **a**,**c**, is disordered due to the absence of the [2Fe–2S] cluster. The DNA is represented by crimson and red lines. The apo-IscR fold is shown in grey in **c**, with holo-ScNsrR depicted as green and blue monomers. The red dashed arrows indicate how the C93–C99 turn displaces the α3 RH region by about 2.5 Å relative to its counterpart in IscR. The double-headed solid red arrow shows a potential clash between the putative C92–C98 turn and the RH as oriented in the apo-IscR structure. ScNsrR Cys93 and Cys99 Cα's are depicted as yellow disks and [4Fe–4S] cluster atoms are shown as small red-brown and yellow spheres. The predicted interaction between Lys95 and the phosphate group of A15 in a holo-IscR-DNA complex is indicated by double-headed green arrows. HTH motif: helix-turn-helix DNA recognition motif; Wing: small groove recognition loop. See also Supplementary Fig. 5. In **d** 3CA-ScNsrR is depicted in grey and holo-ScNsrR in semi-transparent green and blue.

Accessible surface and secondary structure elements of the refined NsrR structure were determined with the dssp program^40^, related structures were superimposed by secondary structure with COOT and this program was also used to visualize anisotropic domain motions (Fig. S2). A cavity map was calculated with the in-house developed program cavenv, as incorporated in the CCP4 package^34^. Compilations of all the programs mentioned above were provided by SBGrid^41^. Figures 2, 4, 5 and , and Supplementary Figures 1,3,5 and 11 were prepared with Molscript^42^ or Bobscript^43^ and rendered with Raster3D (ref. 44).

### Data availability

The coordinates and structure factors have been deposited in the Protein Data Bank under the accession codes 5N07 and 5N08. The PDB accession code 4HF1 corresponding to the EcIscR-DNA complex was used in this study. The UniProt accession codes Q9L132, Q9L131 were also used in this study. All other data are available from the corresponding authors on reasonable request.

## Additional information

**How to cite this article:** Volbeda, A. *et al*. Crystal structures of the NO sensor NsrR reveal how its iron-sulfur cluster modulates DNA binding. *Nat. Commun.* **8,** 15052 doi: 10.1038/ncomms15052 (2017).

**Publisher's note:** Springer Nature remains neutral with regard to jurisdictional claims in published maps and institutional affiliations.

## Supplementary Material

Supplementary Information Supplementary figures and supplementary methods.

## Figures and Tables

**Table 1:** Data collection and refinement statistics.

**Crystal**	1*	**2**	**3**	**4**†
	**Holo-ScNsrR**	**Holo-ScNsrR**	**3CA-ScNsrR**	**3CA-ScNsrR**
*Data collection*				
Space group	P6_5_22	P6_5_22	P3_2_21	P3_2_21
Cell dimensions				
*a*, *b*, *c* (Å)	79.1, 79.1, 93.5	78.8, 78.8, 93.3	159.5, 159.5, 76.6	162.1, 162.1, 76.7
*α*, *β*, *γ* (°)	90, 90, 120	90, 90, 120	90, 90, 120	90, 90, 120
Resolution (Å)‡	38.6–2.6 (2.69–2.6)	39.4–1.95 (2.02–1.95)	43.1–3.9 (4.04–3.90)	39.9–4.0 (4.15–4.01)
*R*_merge_	0.091 (2.321)	0.036 (2.171)	0.157 (2.274)	0.166 (2.229)
*cc*1/2	0.998 (0.501)	0.998 (0.509)	0.999 (0.164)	0.998 (0.152)
*I*/σ*I*	22.7 (1.5)	21.0 (1.0)	9.7 (0.9)	10.0 (0.8)
Completeness (%)	100.0 (99.8)	99.8 (99.0)	98.7 (96.8)	99.2 (97.8)
Redundancy	21.5 (20.4)	8.4 (8.9)	7.8 (7.8)	8.0 (7.0)
				
*Refinement*				
Resolution (Å)		30.0–1.95	43.1–3.9	
Reflections (work/free)		12,307/671	10,288/554	
*R*_work_/*R*_free_		19.5/22.7	27.0/27.7	
N_protein_/asymmetric unit		1	5	
No. of atoms				
Protein		1,016	4,364	
[4Fe–4S]/SO_4_/Cl		8/5/1	0	
Water		31	0	
Average *B*-factors				
Protein		95.1	214.7	
[4Fe–4S]/SO_4_/Cl		71.5/125.6/106.3		
Water		82.2		
R.m.s.d.				
Bond lengths (Å)		0.012	0.005	
Bond angles (°)		1.5	0.87	

^*^Used for phasing by single-wavelength anomalous dispersion (SAD).

^†^used for density modification with multi-crystal averaging (see Methods).

^‡^values in parentheses are for the highest-resolution shell.
