# Supplementary material for: Crystal structures of the NO sensor NsrR reveal how its iron-sulfur cluster modulates DNA binding
Source: Nat Commun. 2017 Apr 20;8:15052. doi: 10.1038/ncomms15052 (PMC5411485; doi:10.1038/ncomms15052)
Supplement: Supplementary Information — Supplementary figures and supplementary methods. [file ncomms15052-s1.pdf]

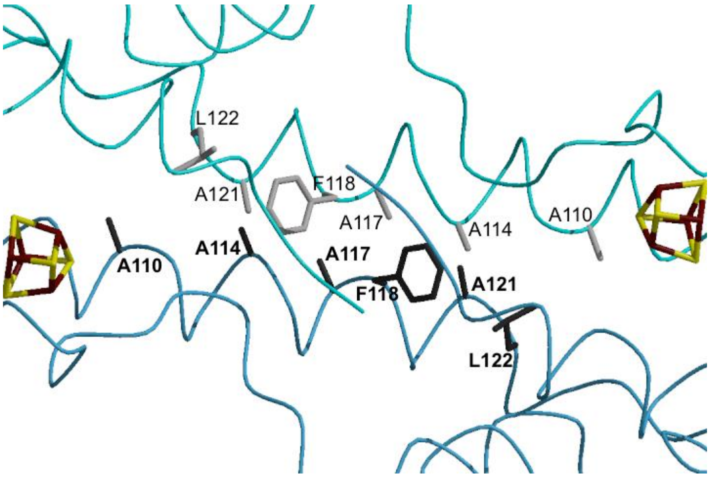

Supplementary Figure 1. Hydrophobic ScNsrR dimer interface.

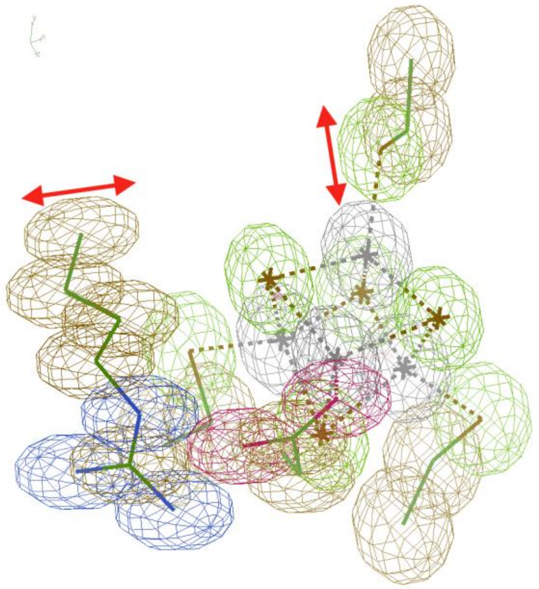

Supplementary Figure 2. Anisotropic temperature factors (obtained from TLS refinement) corresponding to the [4Fe-4S] cluster region of ScNsrR. The arrows indicate the expected different directions of atomic motions of the thiolate ligands of the [4Fe-4S] cluster in one monomer (top right) and of Asp8 and Arg12 in the other (bottom left).

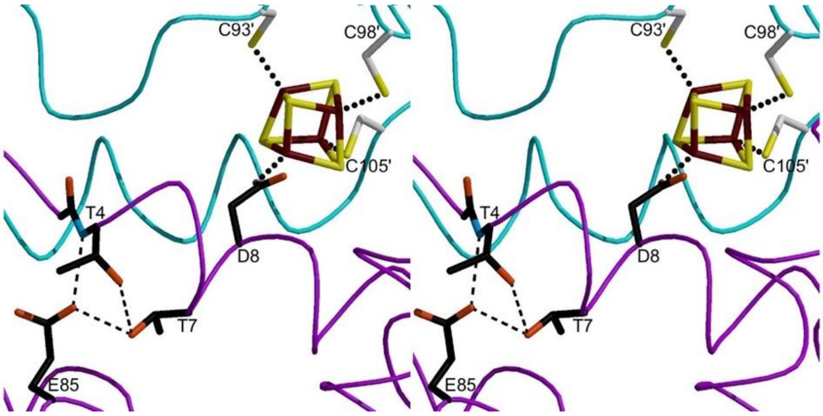

Supplementary Figure 3. Stereo view of the environment of Glu85, which stabilizes the N-terminal segment of ScNsrR through two hydrogen bonds. Atoms are colored as in **Fig. 2B**. Glu85 forms a hydrogen bond with the main chain N of Thr4 and the Oγ of Thr7. Removing these interactions by replacing Glu with Ala at this position is likely to render the N-terminal segment, including Asp8, more flexible. This, in turn, could disrupt the interaction of Asp8 with the [4Fe-4S] cluster from the other monomer causing instability.

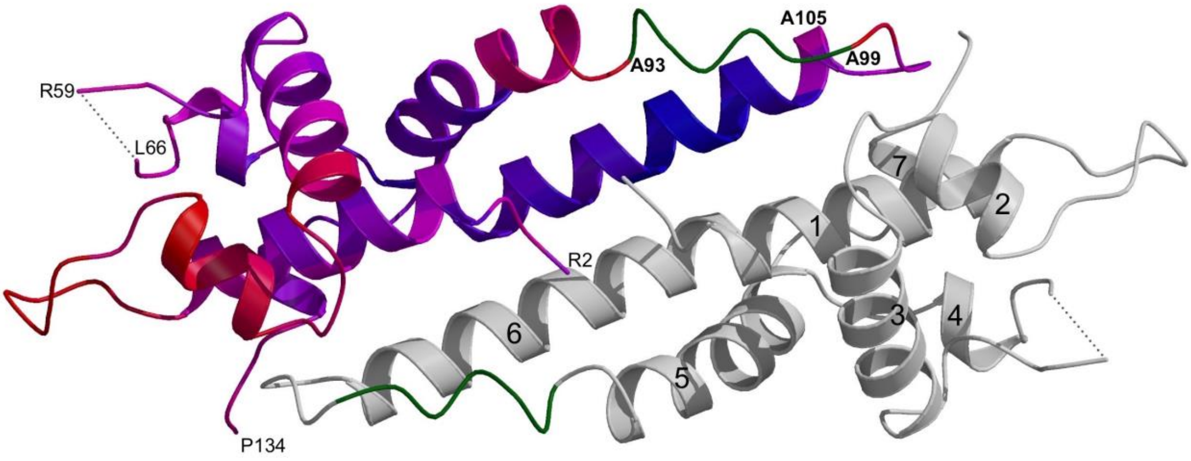

Supplementary Figure 4. Ribbon representation of the apo 3CA-ScNsrR structure. The top monomer is colored from blue to red according to increasing B-factors (from 145.2 Å^2 to 290.4 Å^2). In the bottom monomer (gray), helices are sequentially numbered. The positions of the three mutated residues (Ala93, Ala199 and Ala105) are labeled in bold. The significantly disordered A93-A99 loop is colored in green. Compared to the structure of holo-ScNsrR, the loss of the cluster has induced a lengthening of α-helix 5 of almost two turns, α-helix 6 starts at residue 104 instead of residue 106 and there is a large rearrangement of the C-terminal region following α-helix 7, with residues 135-44 becoming unresolved in the electron density map (see also **Figures 2a and 6d**). The conformation of the Cys93-Cys99 loop in the apo structure is very different from the one in holo-ScNsrR.

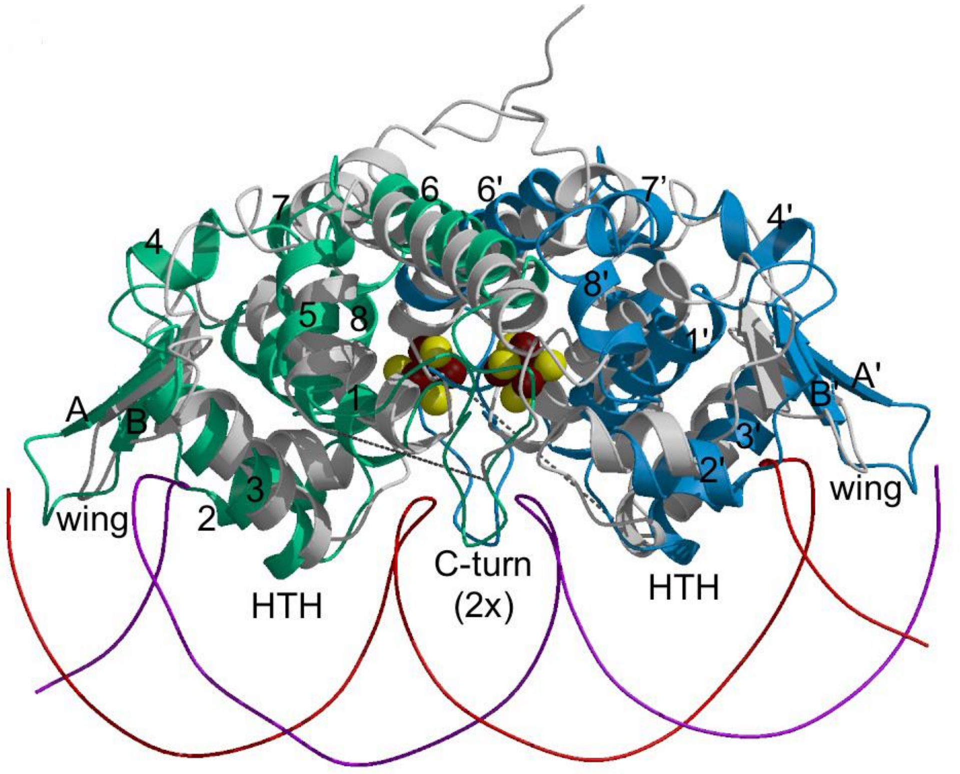

Supplementary Figure 5. Superposition of the EcIscR-DNA complex with ScNsrR (see also **Fig. 6c**) with postulated DNA-binding regions of ScNsrR. HTH: helix-turn-helix region bound at the DNA major groove; wing loop bound at the DNA minor groove; C-turn: inter-cysteine C_{93}EGDNPC_{99} turn.

a) Codon optimized *nsrR* gene sequence

CAT **ATG** CGG TTG ACG AAG TTC ACC GAC CTG GCG CTG CGT TCG CTC ATG CGC
CTG GCG GTC GTG AGA GAC GGT GAC GAA CCA CTG GCC ACC CGA GAG GTG GCC
GAG GTC GTG GGG GTG CCG TAC ACG CAC GCG GCG AAG GCC ATC ACC CGC CTG
CAG CAC CTG GGT GTG GTG GAG GCG CGA CGC GGT CGC GGC GGC GGG CTG ACG
CTG ACC GAC CTG GGC CGG CGC GTC TCC GTG GGC TGG CTG GTG CGT GAA CTC
GAG GGC GAG GCC GAG GTG GTC GAC TGC GAG GGC GAC AAC CCC TGC CCG CTG
CGC GGG GCC TGC CGG CTG CGG CGT GCG CTG CGC GAC GCC CAG GAG GCG TTC
TAC GCG GCA CTC GAC CCA CTG ACC GTG ACC GAC CTG GTG GCC GCA CCG ACC
GGC CCG GTT CTG CTC GGC CTG ACG GAC CGC CCC TCG GGA AAG CTT

b) Translated NsrR protein sequence

MRLTKFTDLALRSLMRLAVVRDGDEPLATREVAEVVGVPYTHAAKAITRLQHLGVVEARR
GRGGGLTLTDLGRRVSVGWLVRELEGEAEVVDCEGDNPCPLRGACRLRRALRDAQEAFYA
ALDPLTVTDLVAAPTGPVLLGLTDRPSG**KL**AAALEHHHHHH

Supplementary Figure 6. Codon optimized sequence of ScNsrR. (a) DNA sequence showing the *nsrR* translational start codon (in bold) and the 5'-NdeI and 3'-HindIII restriction sites (underlined). Highlighted codons were altered for the construction of site-directed variants. (b) Translation of nucleotide sequence shown in (a). Changes to the *nsrR* sequence are in bold. The pGS21a-derived His-tag is underlined.

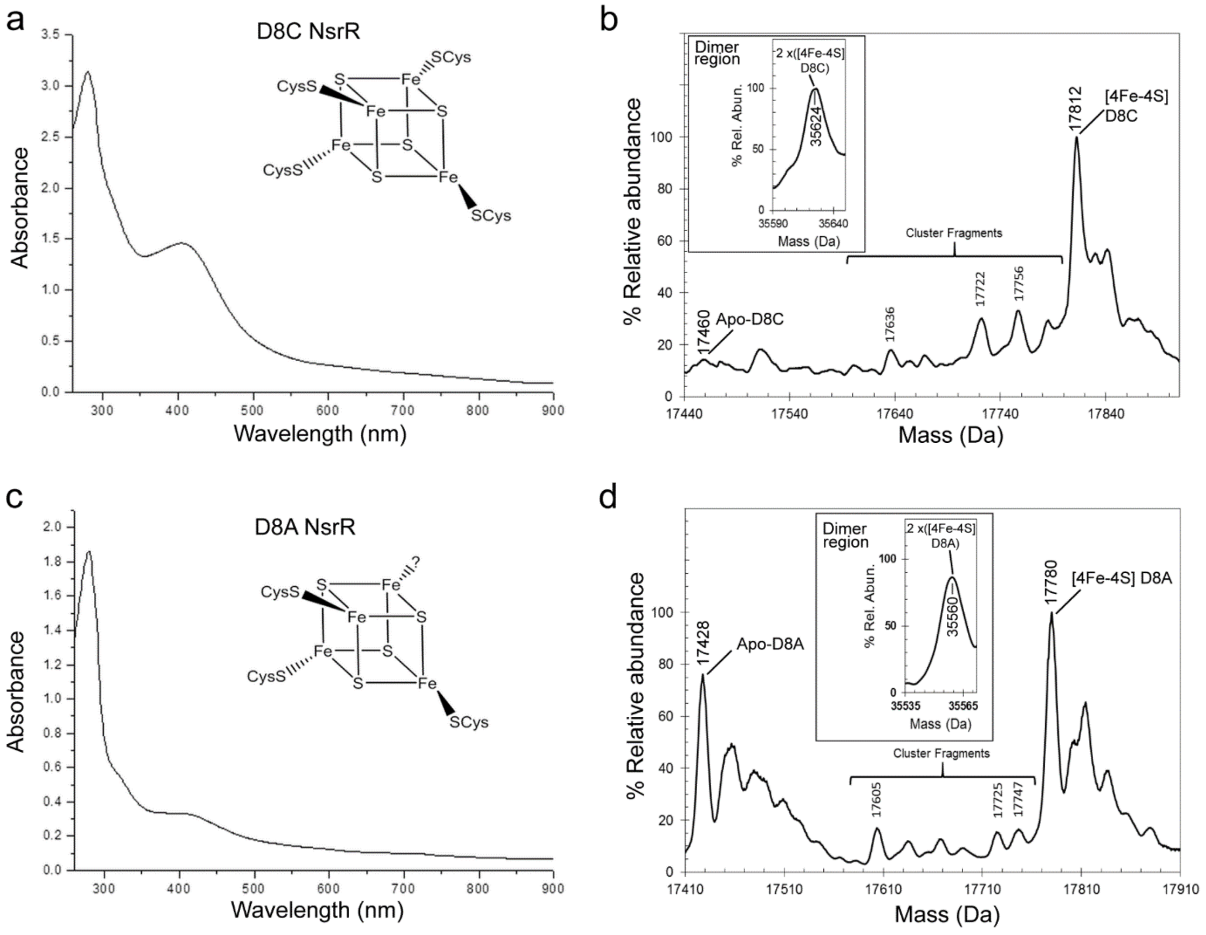

Supplementary Figure 7. Cluster-binding properties of D8C and D8A ScNsrR. (a) and (c) UV-visible absorbance spectra of as isolated D8C and D8A NsrR, as indicated, showing characteristic maxima at 412 nm. (b) and (d) Deconvoluted ESI-MS of D8C and D8A ScNsrR, respectively, under non-denaturing conditions. The data reveal apo and holo forms of the proteins; for D8C ScNsrR, there is very little of the apo form. Observed masses for the apo forms are 17,460 Da (predicted 17,462 Da) for D8C and 17,428 Da (predicted 17,430 Da) for D8A, consistent with the presence of a single disulfide bond in each. The [4Fe-4S] cluster forms are at 17,812 Da (predicted 17,812 Da) for D8C and 17,780 Da (predicted 17,780 Da) for D8A. A number of other minor cluster species are also observed in each spectrum, along with salt adducts of apo and cluster-bound proteins. Inset in (b) and (d) are deconvoluted spectra in the ScNsrR dimer region, with [4Fe-4S] D8C ScNsrR at 35,624 Da (predicted 35,624 Da) and [4Fe-4S] D8A NsrR at 35,560 Da (predicted 35,560 Da). The solution dimer form is susceptible to dissociation (into monomers) during ionization.

a)

Forward	5′- ACACTCGACCCACTGACC -3′
Reverse	5′- TGGGCGTCGAAGAGCTTG -3′

b)

5′-G**CACTCGACCCACTGACC**GTGACCGACCTGGTGGCCGCACCGACCGGCCCGGTTCTGCTC-3′
FORWARD→

5′-GGCCTGACGGACCGCCCCTCGGGATGACGGGCGGCGGCCCCCTGAGGCCGTCGAGCTGTG-3′

5′-GCCT**AAAACACGAATATCATCTACCAATT**AAGGAGTCGCTGTGCTCTCCGAACAGTCCGT-3′
NsrR site

5′-TCCCGTGGTCCGAGCCACCCTCCCCGCCGTCGGAGCGGCCATCGGTGACATCGCCGCCCT-3′

5′-GTTCTACCG**CAAGCTCTTCGACGCCCA**CCCGGAGCTGCTGCGGGACCTGTTCAACCGGGG-3′
←REVERSE

Supplementary Figure 8. Generation of labelled ScNsrR EMSA probe. (a**)** PCR primers (modified with 6-FAM; Integrated DNA technologies) were used to create the ScNsrR EMSA probe. (b**)** Upstream region of *hmpA1* (SCO7428; UniProt accession code Q9L131), showing PCR amplified sequence (underlined). The ScNsrR binding site and PCR primer hybridization sites are shown in bold.

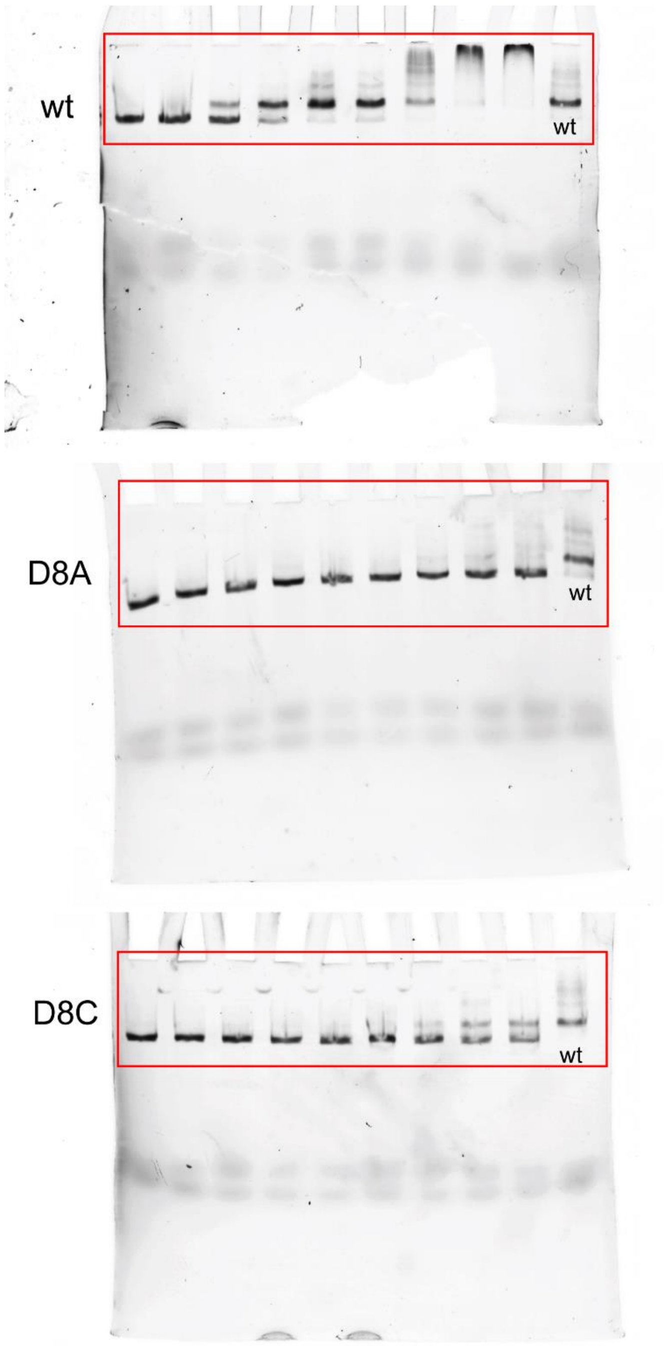

Supplementary Figure 9. Uncropped images for EMSA experiments depicted in **Fig. 4**. Red boxes show approximate images used for presentation in that figure.

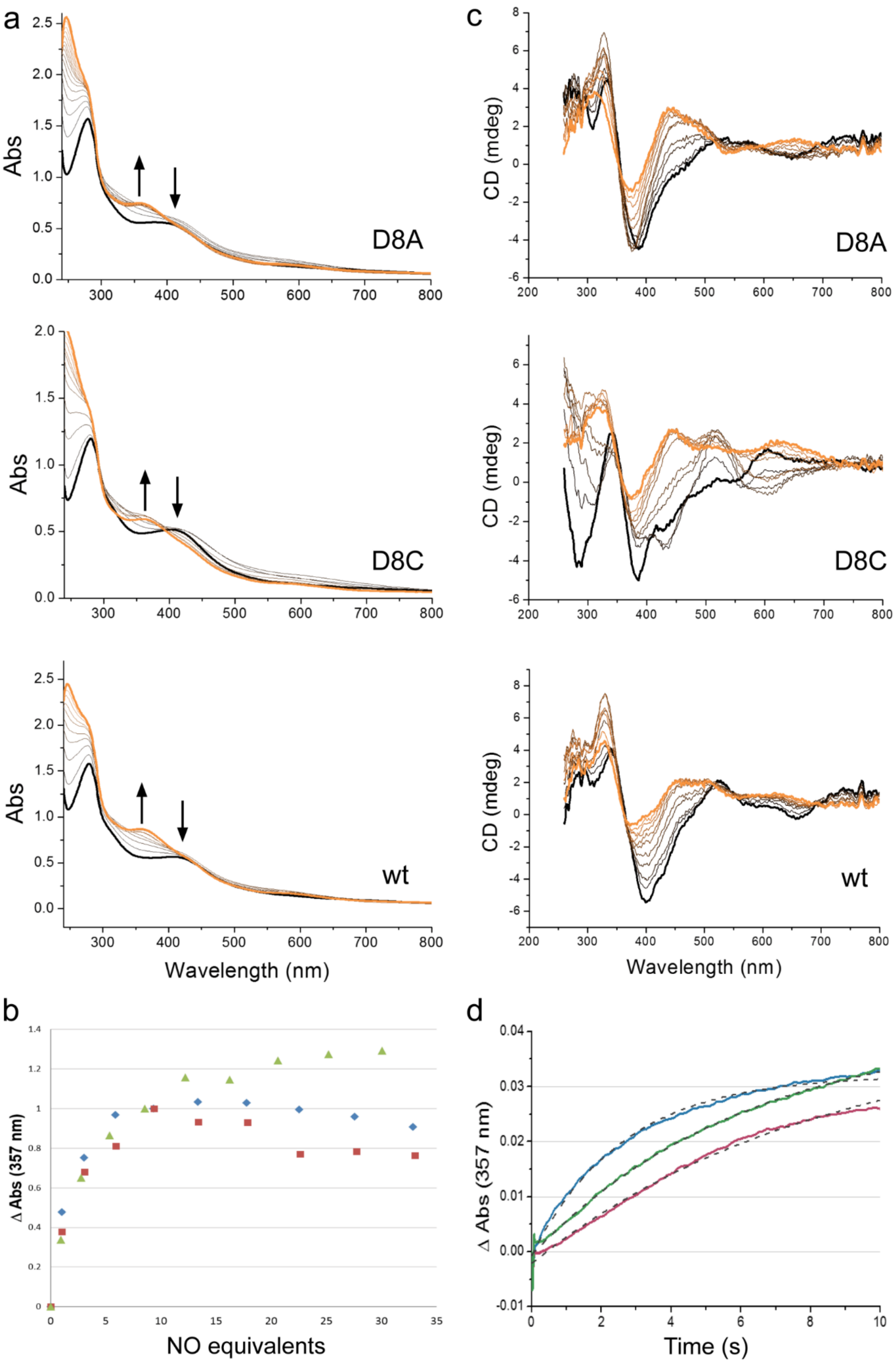

a
Abs
D8A
D8C
wt
Wavelength (nm)
c
CD (mdeg)
D8A
D8C
wt
Wavelength (nm)
b
Δ Abs (357 nm)
NO equivalents
d
Δ Abs (357 nm)
Time (s)

Supplementary Figure 10. Spectroscopic and kinetic characterization of D8 variants of ScNsrR D8A, D8C, and wild type (wt). (a) UV-visible absorbance titration of protein with increasing concentrations of NO. The data are similar for each protein, showing loss of the band at 412 nm and formation of a band at 357 nm. (b) Plots of $\Delta A_{357\ nm}$ as a function of NO per cluster for wild type (green), D8A (blue) and D8C (red) ScNsrR, showing effectively complete formation of products at between 8 and 12 NO molecules per cluster. (c) CD analysis of cluster nitrosylation. The initial spectra (in black) are similar for D8A and wild type ScNsrR proteins, but that for D8C is distinct, with a large negative band at 290 nm, no clear band at 510 nm, and additional features at 420 nm and 600 nm. Changes in the CD observed upon reaction with NO for the D8A variant match those for wild type ScNsrR, i.e., increases at 450 nm and 380 nm, and a rise and then fall at 330 nm (to give final spectrum in orange). For D8C, the observed changes upon reaction with NO are also distinct, with initial decrease in intensity at 600 nm and 430 nm, a sharp increase at 510 nm, and rapid loss of the negative band at 290 nm at under 2 NO per cluster, followed by increases in intensity at 620 nm, 440 nm, 380 nm, and 320, and a decrease at 510 nm. Importantly, the final spectrum (orange) is strikingly similar to that of D8A and wild type ScNsrR. (d) Stopped-flow measurements of $\Delta A_{357\ nm}$ with time following reaction of ScNsrR proteins (wild type, green; D8A, blue; D8C, red) with 116 equivalents of NO. Fitting of the data with a single exponential (broken lines) gave observed rate constants of k_{obs} = 0.37 ± 0.006 s^{-1} for D8A, 0.15 ± 0.004 s^{-1} for wild type and 0.12 ± 0.005 s^{-1} for D8C ScNsrR proteins.

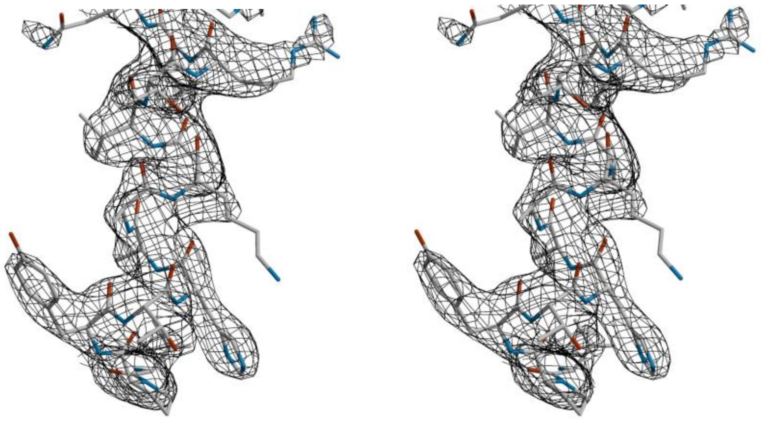

Supplementary Figure 11. Stereo image of the ($2mF_o$-DF_c) 1.95 Å resolution electron density map around helix 3 for holo-ScNsrR. Some exposed side chains are disordered.

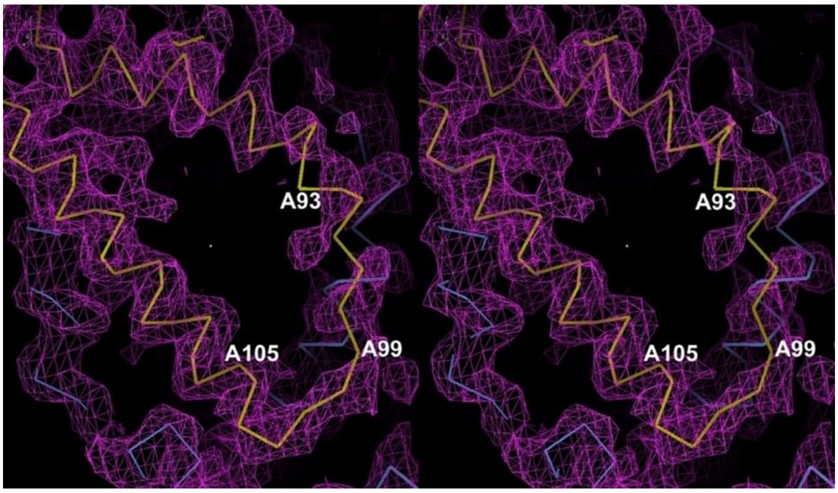

Supplementary Figure 12. Stereographic view of the 10-fold averaged 3.9 Å resolution (2Fo-Fc) electron density map of apo 3CA-NsrR depicting the flexible region between Ala93 and Ala99. See **Methods** for details.

Supplementary Methods

Generation of plasmids encoding C-terminally His-tagged NsrR. The gene sequence of *S. coelicolor nsrR* (SCO7427; UniProt accession code Q9L132) was codon optimized for expression in *E. coli* and synthesized with 5'-NdeI and 3'-HindIII restriction sites by GenScript, see **Supplementary Figure 6**. The synthetic gene was sub-cloned (using NdeI/HindIII) into pGS21a (GenScript) to give wild type ScNsrR with a non-cleavable C-terminal His-tag. Variants (D8A, D8C and C93A/C99A/C105A or 3CA) of ScNsrR were generated by GenScript, by replacement of the relevant codon as follows: GAC to GCC (D8A); GAC to TGC (D8C); TGC to GCC (C93A, C99A, C105A).
